# Supplementary figures and images for: Identification, purification and characterization of mast cells in murine liver fibrosis: optimized methods, expression signatures and correlation with severity
Source: Front Immunol. 2026 Jul 14;17:1852344. doi: 10.3389/fimmu.2026.1852344 (PMC13407189; doi:10.3389/fimmu.2026.1852344)

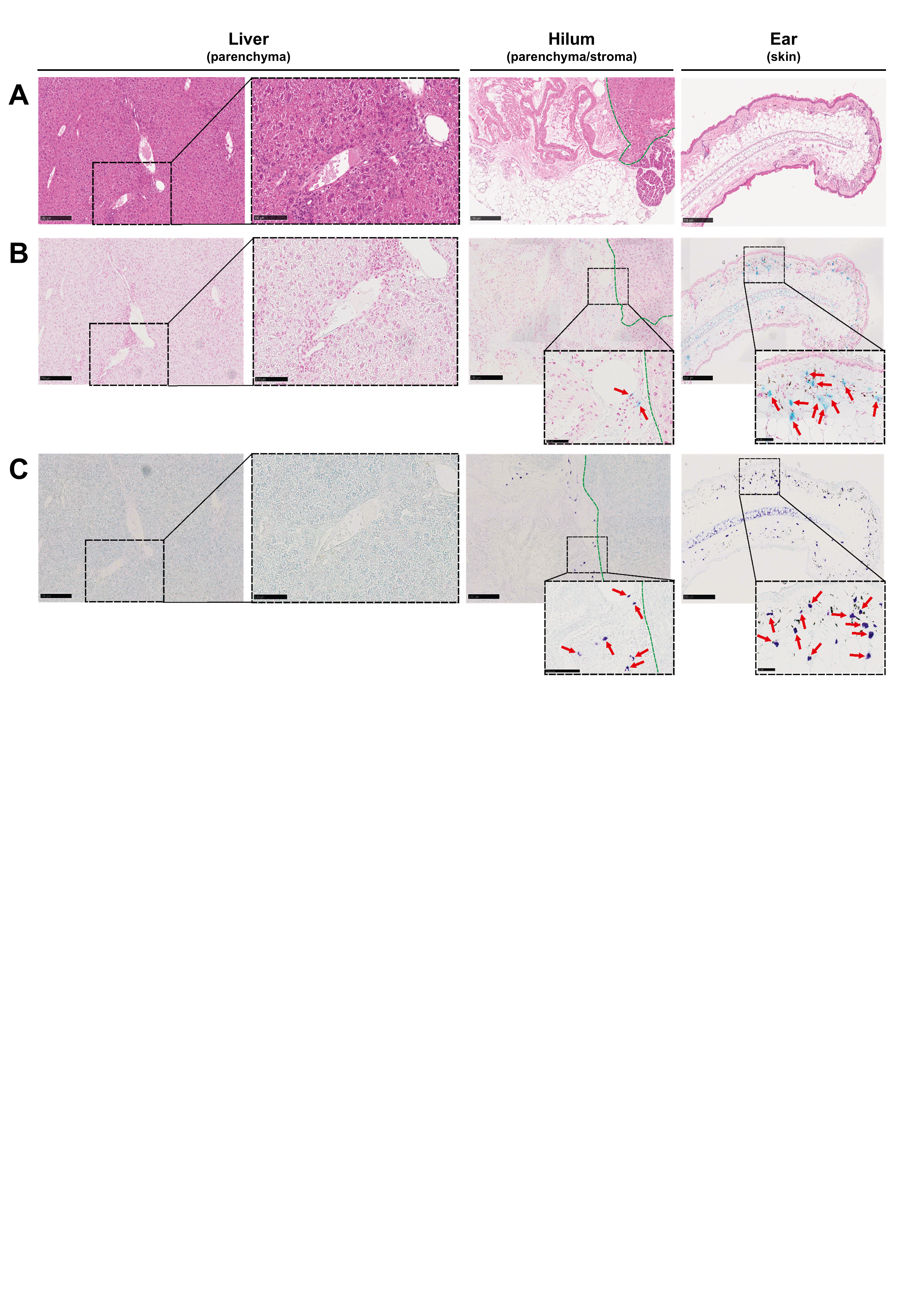

Supplement: Supplementary Figure 1 — MC-specific immunohistological staining methods established on human tissue samples are not applicable in murine liver. (A-C) Formalin fixed, paraffin embedded serial tissue sections of fibrotic murine liver (Mdr2-/-), the hilum area including parenchyma and stromal tissue (separated by a green dashed line) and skin as control tissue (mouse ear) were stained by (A) hematoxylin and eosin, (B) Alcian blue or (C) Toluidine blue to visualize MCs. Stain-positive MCs were exclusively found in control tissues (stroma and skin) and are highlighted by red arrows in enlarged image details. [file Image1.tif]

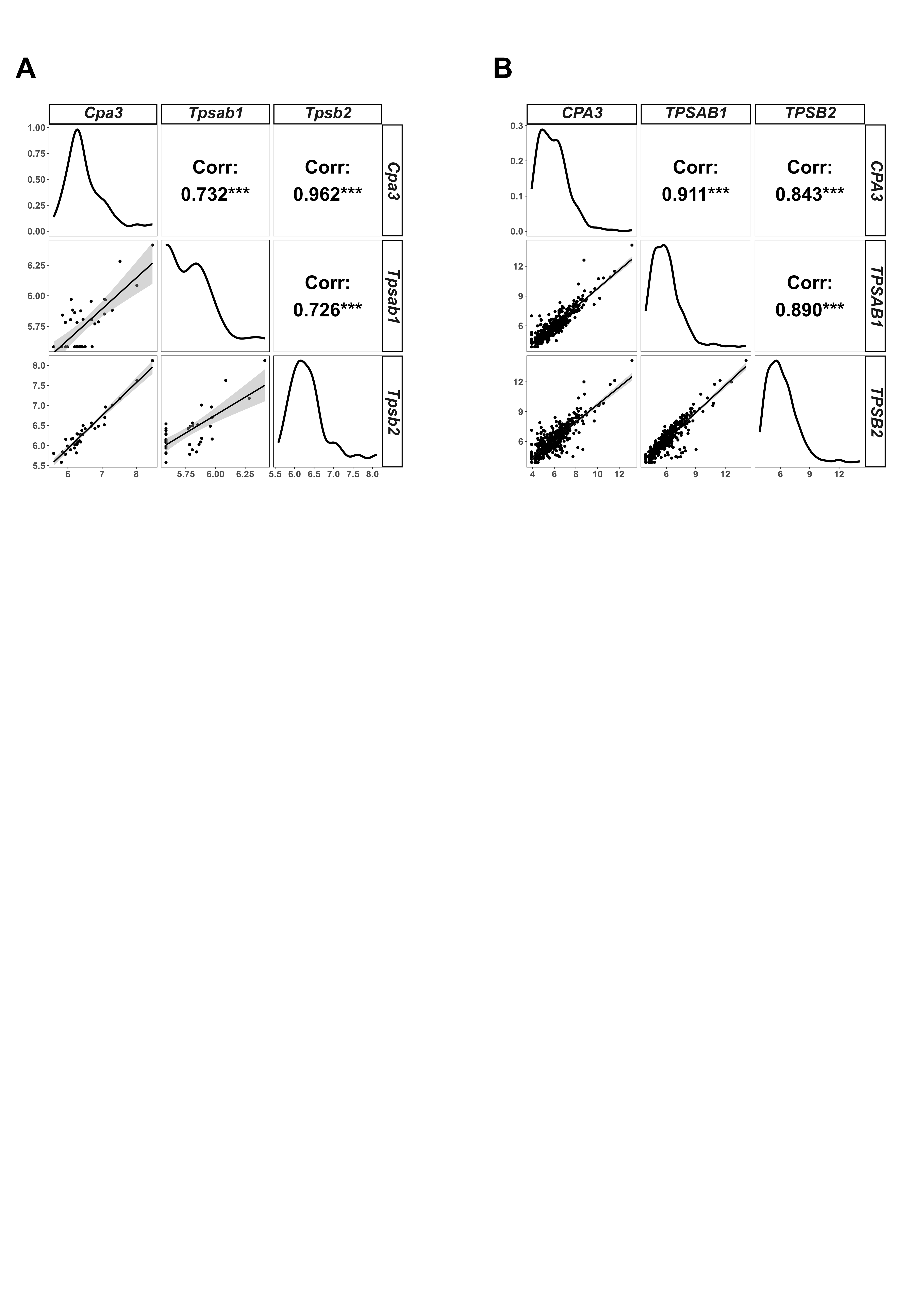

Supplement: Supplementary Figure 2 — Expression of MC markers Cpa3, Tpsab1 and Tpsb2 correlate with each other in murine liver fibrosis and in liver samples from HCC patients. (A) Figure related to Figure 3. VST normalized gene expression data for Cpa3, Tpsab1 and Tpsb2 in murine CCl4-induced liver fibrosis was extracted from the data set GSE167216. The Pearson correlation matrix for Cpa3, Tpsab1 and Tpsb2 is shown. The distribution of expression values is displayed for each gene on the diagonal. In scatter plots below the diagonal, individual measurements of gene expression are plotted against each other, with a regression line and confidence interval (gray area). Pearson correlation coefficients with significance levels are provided in the fields above the diagonal. (B) Figure related to Figure 4. VST normalized gene expression data for CPA3, TPSAB1 and TPSB2 in human HCC samples was extracted from the TCGA-LIHC dataset and displayed as Pearson correlation matrix as in (A). ***p ≤ 0.001. [file Image2.tif]

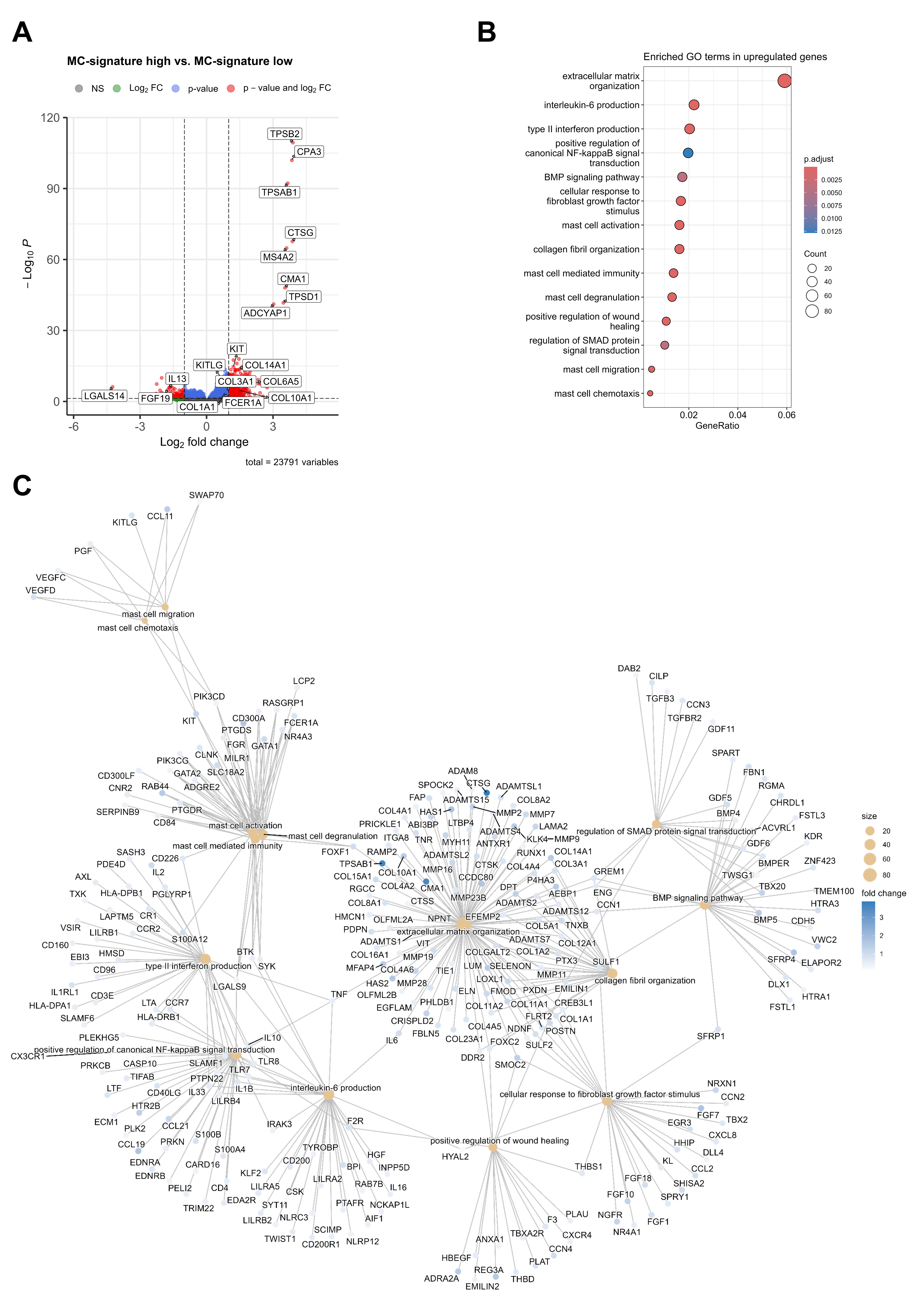

Supplement: Supplementary Figure 3 — High MC signature expression in HCC is associated with increased expression of inflammation- and fibrosis-associated genes. (A) Volcano plot showing differential up- and down-regulated genes between HCC samples with high or low MC gene signature expression. Log2 -fold changes are given on the x-axis while -log10 p-values (adjusted p-values) are shown on the y-axis. The horizontal dashed line indicates the significance level p = 0.05, and vertical dashed lines indicate a 2-fold change of gene expression (log2 (1)). Selected genes are annotated by arrows. (B) Dot plot showing selected enriched gene ontology terms (GO) in up-regulated genes in patients with high expression of MC gene signature. (C) Network plot of enriched GO terms shown in (B) highlighting upregulated genes and their intersection between different gene ontology terms. [file Image3.tif]

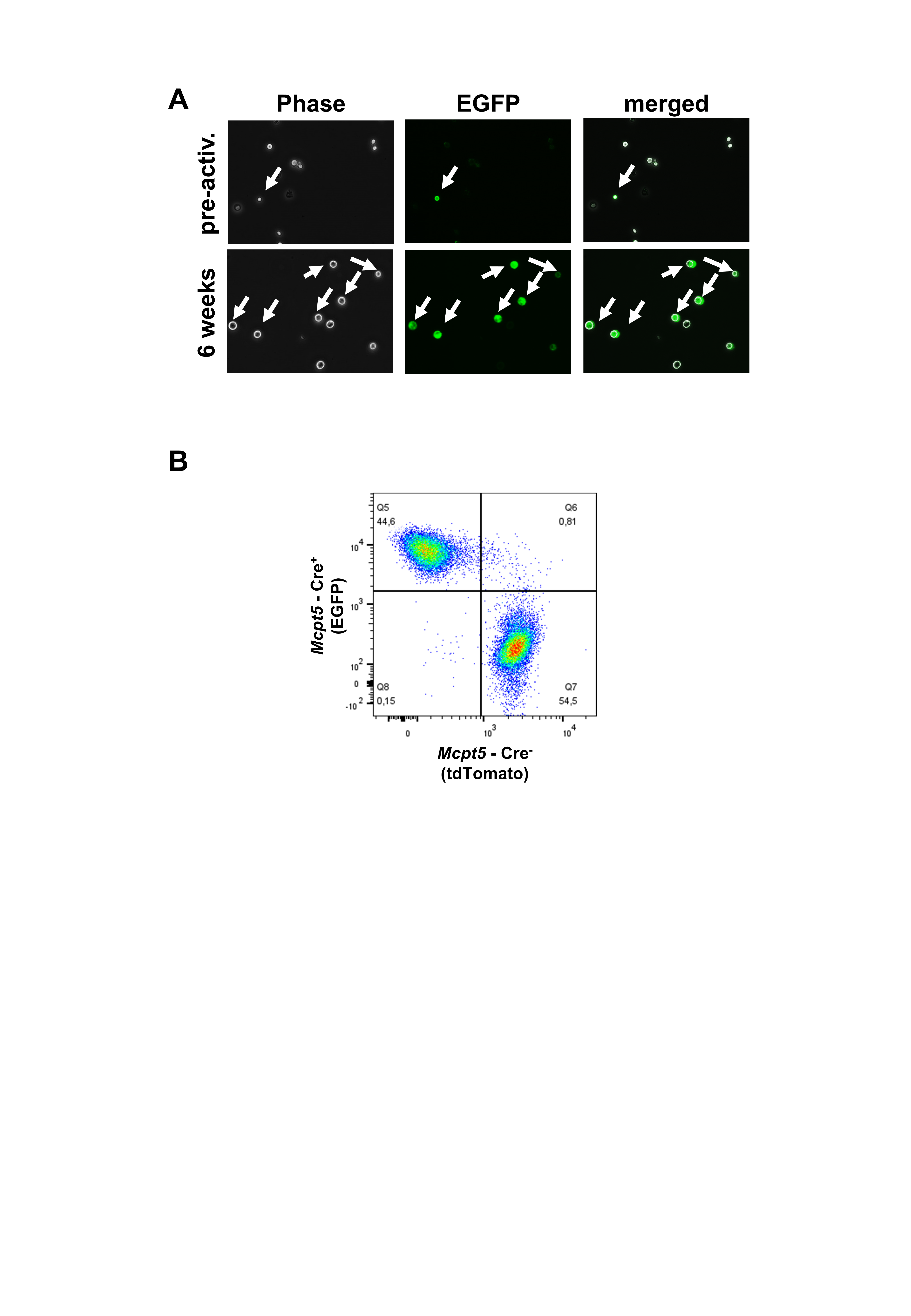

Supplement: Supplementary Figure 4 — Proof of functionality of mT/mG; Mcpt5-Cre reporter mice. (A) BMMCs were generated from mT/mG; Mcpt5-Cre reporter mice (compare Figure 5A) and imaged before (pre-active) and after six weeks of activation with IL-3 to assess EGFP expression. (B) BMMCs generated from mT/mG; Mcpt5-Cre reporter mice were indirectly analyzed for their expression of Mcpt5-Cre by FACS. CD45+ single cells were characterized as Cre+ (EGFP+ tdTomato-) or Cre- (EGFP- tdTomato+). [file Image4.tif]

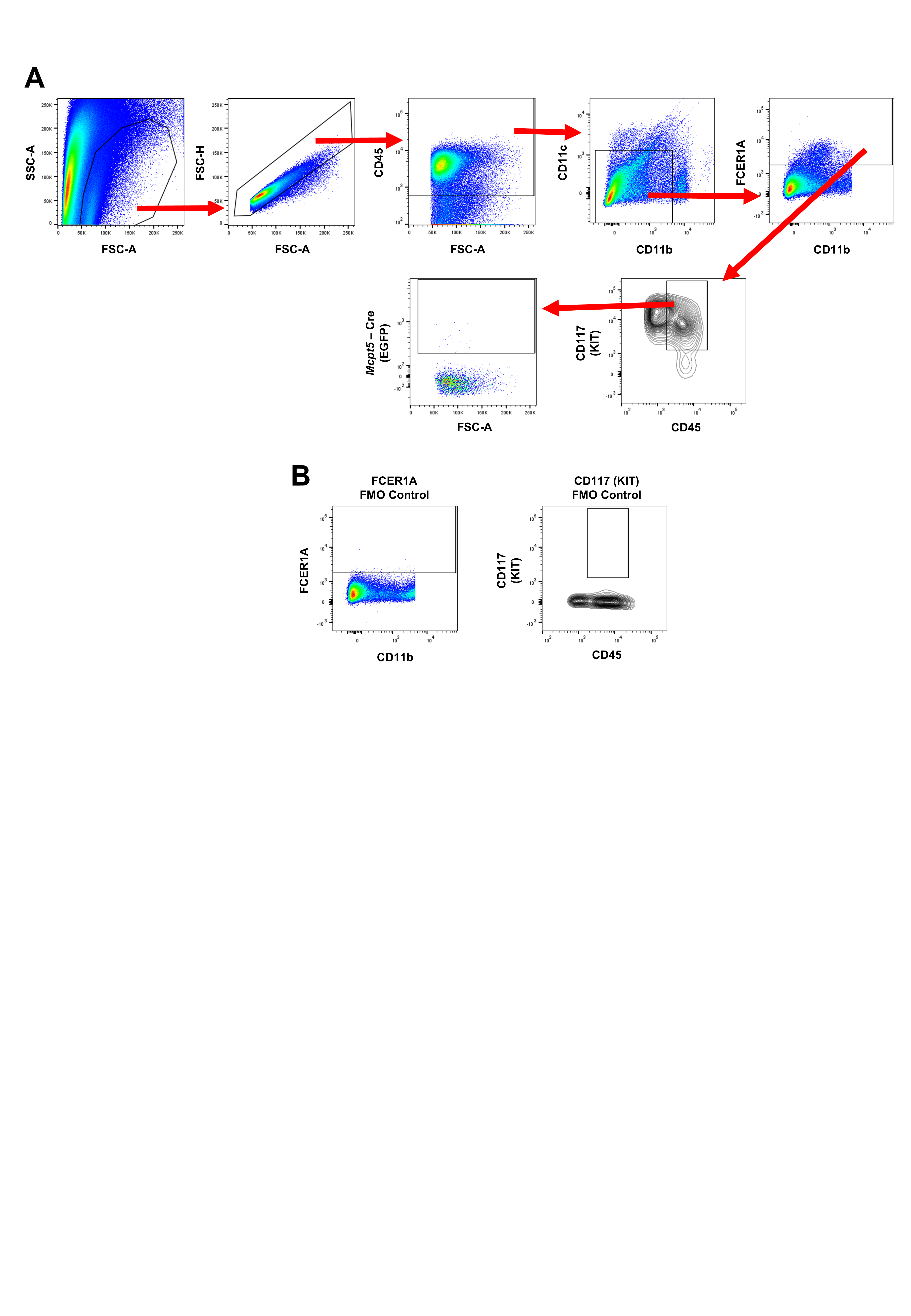

Supplement: Supplementary Figure 5 — FACS gating strategy to detect and purify primary MCs from murine liver. (A) Liver cells were initially selected based on size and granularity (FSC-A vs. SSC-A) and then doublets were removed (FSC-A vs. FSC-H). The immune cell population (CD45+) was further narrowed down to CD11c- and CD11blow-intermediate cells and MCs were gated as FCER1A+ and CD117+ CD45high. MCs isolated from Mcpt5 - reporter mice were additionally characterized by their expression of Mcpt5 - Cre as determined in the EGFP channel. (B) Fluorescence Minus One (FMO) control lacking FCER1A or CD117 antibodies. [file Image5.tif]
